# Posterior Tibial Nerve Stimulation With versus Without Mirabegron: A Randomized Controlled Trial

**DOI:** 10.1007/s00192-024-05835-y

**Published:** 2024-08-05

**Authors:** Russell F. Stanley, Isuzu Meyer, Christina T. Blanchard, Holly E. Richter

**Affiliations:** Division of Urogynecology and Reconstructive Pelvic Surgery, University of Alabama at Birmingham, Bldg. 176F, Suite10382, 619 19th, Street South, Birmingham, AL 35249 USA

**Keywords:** Mirabegron, Nocturia, Overactive bladder, Posterior tibial nerve stimulation, Urinary frequency, Urinary incontinence, Urgency urinary

## Abstract

**Introduction and Hypothesis:**

To compare change in urgency urinary incontinence episodes (UUIEs) in women undergoing posterior tibial nerve stimulation (PTNS) plus mirabegron versus PTNS plus placebo for the treatment of refractory urgency urinary incontinence (UUI). The primary hypothesis was that combination therapy is superior to monotherapy.

**Methods:**

A randomized controlled trial was performed in individuals identifying as female aged ≥ 18 years with UUI symptoms refractory to second-line treatment or who could not tolerate antimuscarinic medications. Both participants and providers were blinded to medication treatment allocation. Participants were randomized (1:1) to PTNS plus mirabegron or PTNS plus placebo. Participants completed a 3-day bladder diary prior to and after 12-week treatment. Validated symptom distress and impact questionnaires were obtained pre- and post-treatment. The primary outcome was change in mean number of UUIEs on a 3-day bladder diary pre- versus post-treatment between arms. Primary and secondary outcomes were analyzed via sample *t* tests.

**Results:**

Fifty-four subjects were randomized, mean ± SD baseline age 56.2±15.6 years and body mass index 35.0±9.4 (kg/m^2^); no differences were noted in any clinical–demographic characteristics. There was a significant difference between arms in mean pre- to post-treatment UUIEs, 9.4±3.9, mirabegron versus 5.3±5.5, placebo (*p*=0.007). Significant differences were found pre- compared with post-treatment in urinary frequency, Overactive Bladder Questionnaire Short Form Symptom Bother and Symptom Health-Related Quality of Life scores.

**Conclusions:**

In subjects undergoing PTNS treatment for refractory UUI and OAB-wet symptoms, the addition of a β-3 agonist produced significant improvement in both objective and subjective overactive bladder symptom outcomes compared with PTNS plus placebo.

## Introduction

Urgency urinary incontinence (UUI) is the complaint of involuntary loss of urine associated with urgency, a common condition that affects between 9 and 31% of women [1–3]. Overactive bladder (OAB), is defined as urinary urgency, usually accompanied by increased daytime frequency and/or nocturia, with urinary incontinence (OAB-wet) or without (OAB-dry), in the absence of urinary tract infection or other detectable disease [4]. This condition presents a significant health care burden costing up to 19 billion US dollars per year [5]. First-line therapy includes behavioral therapy such as caffeine restriction and timed voiding, in addition to pelvic floor muscle therapy (PFMT) and other strategies [6]. Second-line therapy includes medication with either antimuscarinic or β-3 agonist medications [7]. Antimuscarinic medications have long been the mainstay of second-line treatment for OAB/UUI, but have significant side effects, including dry mouth, dry eyes, and constipation [7–9]. In addition, there is growing consensus that anticholinergic medications should be avoided in older women because of increased cognitive impairment caused by antimuscarinic medications [10, 11].

Current advanced bladder therapy options include sacral neuromodulation (SNM), Onabotulinumtoxin A (Botox®) injected into the detrusor muscle during cystoscopy, and percutaneous tibial nerve stimulation (PTNS). Recent interest has peaked with respect to a combination of second-line and advanced bladder therapies [12]. PTNS in combination with antimuscarinic medications has been studied and compared [13–16]. In most of these studies, when PTNS plus placebo was compared with PTNS in combination with active antimuscarinic medication, the active combination therapy was found to be more effective than PTNS plus placebo or an antimuscarinic medication alone. In previous systematic reviews, where PTNS alone was compared with medications (antimuscarinic), no difference was found and each monotherapy was considered to be equally efficacious [17, 18].

The rationale for studying mirabegron in combination with PTNS versus PTNS plus placebo is important, as mirabegron does not have the significant side effects profile that antimuscarinic medications have for cognitive impairment in older patients. Despite some of the side effects that mirabegron can have in older patients, such as the risk of hypertension, palpitations, and interactions with certain beta blockers, it is better tolerated by many patients. The objective of this study was to compare the efficacy of the combined therapy, PTNS plus mirabegron, with PTNS plus placebo, in women with refractory UUI. The hypothesis was that subjects in the PTNS plus mirabegron arm would have a greater change in the number of UUI episodes pre- compared with post-treatment than those in the PTNS plus placebo arm.

## Materials and Methods

A randomized controlled trial in individuals identifying as female aged ≥ 18 years with UUI symptoms refractory to second-line treatments (including both antimuscarinics and/or mirabegron) or who could not tolerate antimuscarinic medications. Institutional Review Board approval was obtained after a rigorous review of the study was completed. Inclusion and exclusion criteria are noted in Table 1. After written informed consent was obtained, participants were randomized to either PTNS plus mirabegron or PTNS plus placebo in a 1:1 fashion using variable sized permuted blocks of 2, 4, and 6. A sequentially numbered sealed envelope was selected to reveal the randomized intervention. This was done by a research team member on the day of the first PTNS treatment. Both participants and providers were blinded to medication treatment allocation. Allocation concealment was ensured by using sequentially numbered, opaque, sealed envelopes.

**Table 1:** Inclusion/exclusion criteria

Inclusion criteria	Exclusion criteria
Patients identifying as female aged **≥** 18 years with refractory urgency urinary incontinence in whom first-line and second-line treatments have failed or who cannot tolerate anticholinergic medications and have been referred for PTNS	Patients with a history of any known or determined urinary retention or urinary tract obstruction
Ability to consent	PVR > 150 ml in the clinic prior to the start of PTNS
Ability to complete all study-related items and interviews	History of bladder augmentation surgery
	Patients who are pregnant or who are suspected to be pregnant
	Uncontrolled hypertension
	Hypersensitivity to mirabegron, such as hypertension, palpitations, constipation, nausea
	Superficial and/or deep skin infection where PTNS intervention is required
	Spinal cord injury of clinically significant neurological disorders known to affect urgency urinary incontinence
	Bleeding diathesis
	Failure of previous advanced bladder therapies such as sacral neuromodulation, PTNS, or Botox
	Pacemaker or implantable defibrillator
	Current use of Interstim sacral nerve stimulator or TENS in the pelvic region, back, or legs
	Coagulopathy
	Chronic swollen, infected, inflamed skin or skin eruptions (e.g., phlebitis, thrombophlebitis, varicose veins) in the region where the PTNS needles or surface electrodes would be placed
	Metal implant in foot/toes near TENS electrode location
	Marked sensory deficit (numbness) of feet or ankles in the region where the PTNS needles or surface electrodes would be placed
	Unwilling to use acceptable form of contraceptive if the participant is of childbearing potential
	Unable or unwilling to complete the 3-day bladder diary
	Visual impairment prohibiting reading the paper diary
	Inability to provide informed consent, complete questionnaires independently, or to attend intervention sessions
	Unable to speak, read, or write in English

*PVR* post-void residual, *PTNS* posterior tibial nerve stimulation, *Botox* onabotulinum toxin, *TENS* transcutaneous electrical nerve stimulation

Participants who were on oral UUI medication therapy discontinued it 2 weeks prior to the initiation of the assigned treatment. One arm of the study received PTNS combined with mirabegron and the other arm of the study received PTNS with a matching placebo. In the PTNS with mirabegron arm, the participant took a 50-mg dose daily for the 12 weeks of the trial. In the PTNS with placebo arm, the participant took a matching placebo daily during the 12-week trial. PTNS was performed as described: utilizing Urgent® PC (Laborie, Portsmouth, NH, USA), the percutaneous approach entails insertion of a 36-gauge needle electrode at a 60° angle approximately 5 cm or three finger breadths cephalad to the medial malleolus and posterior to the tibia. A portable electrical stimulator delivers an adjustable current within the range 0.5–9 mA. The generators were set for a pulse frequency of 20 Hz, with a goal of creating a motor and/or sensory response in the foot. The stimulation sessions lasted for 30 min once per week for 12 consecutive weeks.

Baseline clinical and demographic characteristics were collected, including age, body mass index (BMI), race, ethnicity, education level, income level, smoking status, menopausal status, Charlson Comorbidity Index [19, 20], and the Pain Catastrophizing Scale [21]. The primary outcome was change in number of urgency urinary incontinence episodes (UUIEs) over a 3-day bladder diary (completed over 3 consecutive days and nights) pre- to post-treatment. The participants completed a 3-day bladder diary prior to and at the end of the 12-week treatment. The secondary outcomes included urinary frequency, stress urinary incontinence episodes (SUIEs), nocturia, and total pad use on the 3-day bladder diary. Additionally, validated symptom distress and impact questionnaires were collected, which include the Urogenital Distress Inventory (UDI-6) [22], the Overactive Bladder Questionnaire-Short Form (OAB-q SF) [23], the Incontinence Impact Questionnaire Short Form (IIQ-7) [22] questionnaires, as well as adverse events. Adverse events were collected on a data collection sheet at each visit; medication-associated adverse effects such as feeling sick (nausea), constipation, diarrhea, urinary tract infection (UTI), headaches, feeling dizzy, hypertension, and palpitations, and for PTNS, which included bleeding at the stimulation site, lower-extremity swelling, worsening of urinary incontinence, leg cramps, vasovagal response, and a generalized headache.

Based on existing data [14], with the estimated difference in UUIEs of 2.5 UUI episodes (standard deviation ± 3) pre- to post-treatment over a 3-day bladder diary, 27 subjects per group (a total of 54 subjects) provided 80% power at a 2-sided α of 0.05, assuming an attrition rate of 10%.

The primary outcome was analyzed via two-sample *t* tests; Chi-squared tests or Fisher’s exact tests for categorical variables; and paired *t* (intra-arm) or two-sample *t* tests (inter-arm) for continuous variables were used as appropriate. Analysis was per protocol, including all women providing 12-week outcome data. A significance level was set at 0.05. No adjustments were made for multiple comparisons. All analyses were performed using SAS v9.4 (SAS Institute, Cary, NC, USA). The statistical analysis plan was developed by our statistician prior to study recruitment initiation.

## Results

**Fig. 1:**
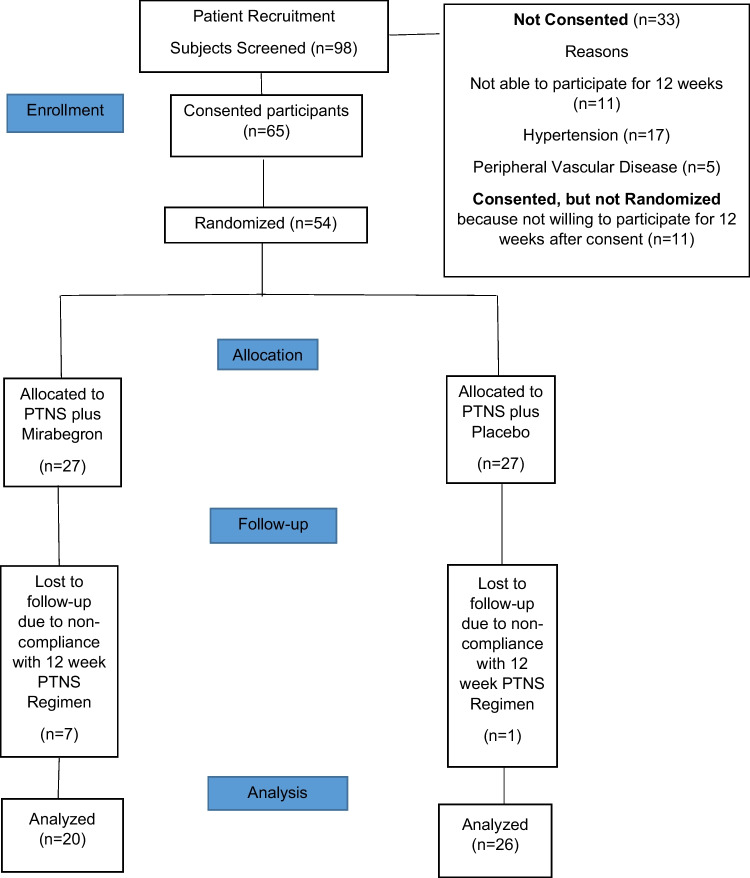
Consolidated standards of reporting trials diagram

From January 2022 to April 2023, a total of 98 subjects were screened, 65 subjects consented and 54 were randomized (Fig. 1). Mean ± SD baseline age 56.2 ± 15.6 and baseline BMI was 35.0 ± 9.4 (kg/m^2^). There were no differences between arms in any clinical–demographic variables noted (Table 2). Eight subjects (7 mirabegron, 1 placebo) did not complete the study owing to noncompliance with 12 weeks of PTNS treatment. Subjects who completed the 12 weeks of PTNS treatment were analyzed per protocol.

**Table 2:** Baseline clinical and demographic characteristics

Characteristics	All (*n*=54)	Mirabegron arm (*n*=27)	Placebo arm (*n*=27)	p*
Age, mean ± SD	56.2 ± 15.6	55.8 ± 16.7	56.6 ± 14.7	0.85
BMI (kg/m^2^), mean ± SD	35.0 ± 9.4	34.3 ± 8.1	35.6 ± 10.7	0.61
Race, *n* (%)				1.00
White	28 (51.9)	14 (51.9)	14 (51.9)	
Black or African American	26 (48.2)	13 (48.2)	13 (48.2)	
Ethnicity, *n* (%)				–
Hispanic	0 (0.0)	0 (0.0)	0 (0.0)	
Not Hispanic or not Latina	54 (100.0)	27 (100.0)	27 (100.0)	
Education, *n* (%)				0.68
Less than high school	2 (3.7)	2 (7.4)	0 (0.0)	
Completed high school or GED	20 (37.0)	10 (37.0)	10 (37.0)	
Completed associate degree	13 (24.1)	7 (25.9)	6 (22.2)	
Completed 4-year degree	8 (14.8)	4 (14.8)	4 (14.8)	
Completed graduate degree	11 (20.4)	4 (14.8)	7 (25.9)	
Income, *n* (%)				0.34
Less than US$20,000	19 (35.2)	12 (44.4)	7 (25.9)	
US$20,000–US$40,000	29 (53.7)	13 (48.2)	16 (59.3)	
US$40,000–US$80,000	6 (11.1)	2 (7.4)	4 (14.8)	
Smoking status, *n* (%)				0.74
No smoking	43 (79.6)	21 (77.8)	22 (81.5)	
Smoking	11 (20.4)	6 (22.2)	5 (18.5)	
Menopausal status, *n* (%)				0.78
Pre-menopausal	19 (35.2)	10 (37.0)	9 (33.3)	
Post-menopausal	35 (64.8)	17 (63.0)	18 (66.7)	
Charlson Comorbidity Index, mean ± SD	2.7 ± 1.9	2.6 ± 2.0	2.8 ± 1.8	0.78
Pain Catastrophizing Scale, mean ± SD	15.2 ± 14.2	14.4 ± 10.6	15.9 ± 17.1	0.70

Data are presented as mean ± SD or *n* (%) where indicated

*Chi-squared tests or Fisher’s exact tests for categorical variables

There was a significant difference between the mirabegron and placebo arms in changes pre- to post-treatment UUIEs (mirabegron arm 9.4 ± 3.9 versus placebo arm 5.3 ± 5.4, *p*=0.007, Table 3). Secondary outcomes revealed significant differences between pre- and post-treatment arms in urinary frequency (mirabegron arm 11.2 ± 6.8, placebo arm 6.1 ± 6.1, *p*=0.012), change in the OAB-q SF Symptom Bother score (mirabegron arm 53.6 ± 30.0, placebo arm 33.3 ± 35.7, *p*=0.048), and change in OAB-q SF Symptom HRQL score (mirabegron arm −32.7 ± 20.5, placebo arm −15.1 ± 19.6, *p*=0.005). No significant differences were noted in other secondary outcomes (Table 3). Significant improvements were noted in all within-arm outcomes, with the exception of the pre- to post-treatment outcome for SUIEs in the placebo arm (*p*=0.14, Table 4).

**Table 3:** Primary and secondary outcome measures for the mirabegron and placebo arms

Outcome measures	Mirabegron arm ∆ (*n*=20)	Placebo arm ∆ (*n*=26)	*p*
Total number of UUI episodes over 3 days	9.4 ± 3.9	5.3 ± 5.4	0.007
Total number of SUI episodes over 3 days	1.4 ± 2.2	1.4 ± 4.6	0.99
Total number of daytime voids over 3 days	11.2 ± 6.8	6.1 ± 6.1	0.012
Total number of nighttime voids over 3 days	4.3 ± 2.6	2.8 ± 2.7	0.07
Total number of pads used over 3 days	6.5 ± 4.9	3.2 ± 6.5	0.06
IIQ-7	29.7 ± 17.4	20.2 ± 17.6	0.07
UDI-6	23.6 ± 12.9	16.3 ± 14.0	0.08
OABq SF Symptom Bother Scale	53.6 ± 30.0	33.3 ± 35.7	0.048
OABq SF HRQL scale	−32.7 ± 20.5	−15.1 ± 19.6	0.005

Data are presented as changes in mean ± SD

*UUI* urgency urinary incontinence, *SUI* stress urinary incontinence, *IIQ-7* Incontinence Impact Questionnaire , *UDI-6* Urinary Distress Index, *OABq SF* Overactive Bladder Questionnaire-Short Form, *HRQL* Health-Related Quality of Life

*Comparison made using sample *t* tests

**Table 4:** Changes in outcome measures baseline to post-treatment within arms

Group	Baseline	Post-treatment	p*
Total number of UUI episodes over 3 days			
Mirabegron arm (*n*=20)	14.9 ± 7.2	5.5 ± 5.0	<0.001
Placebo arm (*n*=26)	16.7 ± 9.2	11.3 ± 8.1	<0.001
Total number of SUI episodes over 3 days			
Mirabegron arm (*n*=20)	3.0 ± 5.5	1.6 ± 3.6	0.011
Placebo arm (*n*=26)	2.5 ± 5.3	1.1 ± 2.2	0.14
Total number of daytime voids over 3 days			
Mirabegron arm (*n*=20)	30.5 ± 10.8	19.3 ± 5.9	<0.001
Placebo arm (*n*=26)	27.3 ± 10.3	21.2 ± 8.2	<0.001
Total number of nighttime voids over 3 days			
Mirabegron arm (*n*=20)	5.3 ± 3.1	1.1 ± 1.5	<0.001
Placebo arm (*n*=26)	5.8 ± 5.1	3.0 ± 3.4	<0.001
Total number of pads used over 3 days			
Mirabegron arm (*n*=20)	9.8 ± 7.4	3.3 ± 4.3	<0.001
Placebo arm (*n*=26)	8.4 ± 12.3	5.2 ± 6.5	0.019
IIQ-7			
Mirabegron arm (*n*=20)	52.7 ± 26.8	23.0 ± 15.8	<0.001
Placebo arm (*n*=26)	54.5 ± 24.6	34.3 ± 28.7	<0.001
UDI-6			
Mirabegron arm (*n*=20)	42.1 ± 19.3	18.5 ± 14.8	<0.001
Placebo arm (*n*=26)	43.4 ± 14.4	27.1 ± 16.0	<0.001
OABq SF Symptom Bother			
Mirabegron arm (*n*=20)	75.0 ± 34.8	21.5 ± 22.6	<0.001
Placebo arm (*n*=26)	73.0 ± 37.5	39.7 ± 37.7	<0.001
OABq SF HRQL			
Mirabegron arm (*n*=20)	47.5 ± 22.5	80.2 ± 17.5	<0.001
Placebo arm (*n*=26)	47.7 ± 26.1	62.8 ± 28.2	<0.001

Data are presented as mean ± SD

*UUI* urgency urinary incontinence, *SUI* stress urinary incontinence, *IIQ-7* Incontinence Impact Questionnaire , *UDI-6* Urinary Distress Index, *OABq SF* Overactive Bladder Questionnaire-Short Form, *HRQL* Health-Related Quality of Life

*Data analyzed using paired *t* tests

**Fig. 2:**
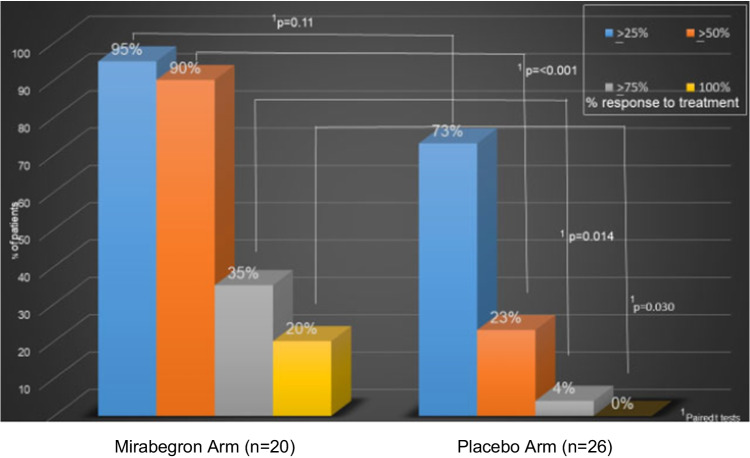
Response rates for reduction in urgency urinary incontinence (*UUI*) episodes pre- to post-treatment. The columns on the *left* indicate the percentage of the population that had ≥25%, ≥50%, ≥75%, or 100% reduction in UUI episodes pre- to post-treatment in the mirabegron arm. The columns on the *right* indicate the percentage of the population that had ≥25%, ≥50%, ≥75%, or 100% reduction in UUI episodes pre- to post-treatment in the placebo arm. The *p* values are based on paired *t* tests, which compare the response rates in the mirabegron arm with those in the placebo arm

There were significant differences in the proportion of patients who had greater than ≥50%, 75%, and 100% reduction in UUI episodes pre- to post-treatment favoring the mirabegron arm compared with the placebo arm (*p*<0.05, Table 5). In the mirabegron arm, 90.0% of subjects had ≥ 50% reduction in UUIEs compared with 23.1% in the placebo arm (*p*<0.001); 20% of patients in the mirabegron arm versus 0% in placebo experienced a 100% reduction in UUIEs pre- to post-treatment (Fig. 2).

**Table 5:** Response rates for reduction in UUI episodes pre- to post-treatment

Responder^a^	Overall (*n*=46)	Mirabegron arm (*n*=20)	Placebo arm (*n*=26)	p*
≥25% reduction in UUI episodes from pre-trial to post-trial	38 (82.6)	19 (95.0)	19 (73.1)	0.11
≥50% reduction in UUI episodes from pre-trial to post-trial	24 (52.2)	18 (90.0)	6 (23.1)	<0.001
≥75% reduction in UUI episodes from pre-trial to post-trial	8 (17.4)	7 (35.0)	1 (3.9)	0.014
100% reduction in UUI episodes from pre-trial to post-trial	4 (8.7)	4 (20.0)	0 (0.0)	0.030

Data are presented as *n* (%)

*UUI* urgency urinary incontinence

*Paired *t* tests

^a^ Reference group = placebo

Adverse events included 3 patients in each arm who experienced UTIs that resolved with antibiotics treatment. Additionally, 3 patients in each arm reported transient leg swelling from PTNS treatments, which resolved spontaneously. All patients who reported adverse events completed the full 12-week PTNS treatment protocol.

## Discussion

This study demonstrated that combined active second-line and advanced bladder combination therapy produced significant reduction in UUIEs pre- to post-treatment compared with advanced bladder therapy and placebo arm. Additionally, active combination therapy produced significantly improved changes in secondary objective and subjective secondary outcomes such as urinary frequency, OAB-q SF symptom bother score, and OAB-q SF HRQL symptom score pre- to post-treatment compared with the placebo arm. Furthermore, the proportion of patients in the active combination therapy arm who had ≥50%, 75%, and 100% reduction in UUIEs pre- to post-treatment was greater than in the placebo arm, with 20% of patients in the active combination therapy arm attaining 100% reduction in UUIEs. These data demonstrate the important consideration of combination therapy.

In previous combination therapy studies, solifenacin or tolterodine were studied in combination with PTNS or stroller afferent neuro-stimulation (SAN) respectively, versus monotherapy [13–16]. In those studies, active combination therapy with an antimuscarinic medication and peripheral neuromodulation produced significant improvement in both objective and subjective overactive bladder symptoms compared with monotherapy or placebo [13–16]. Similar to the current study, these studies reflected a statistically significant reduction in UUI episodes and in urinary frequency in the active combination therapy group compared with monotherapy. Those studies also found statistically significant changes in subjective symptoms measured with validated questionnaires. The findings from those studies are similar to those of the current study in that they make the case for considering combination therapy over monotherapy when treating patients with refractory UUI symptoms. Additionally, those studies had similar sample sizes to the current study and looked at very similar objective outcomes (bladder diary) and symptom-based outcomes via validated questionnaires. The novelty in the current study is the use of the relatively new β-3 agonist instead of the anticholinergic medications in combination with PTNS.

Although antimuscarinic medications have contraindications for patients with impaired gastric motility, urinary retention, and narrow-angle glaucoma, the β-3 agonists do not have such contraindications. There is growing evidence that supports avoiding the use of antimuscarinic medication in older women owing to an increased risk of cognitive impairment [10, 11]. β-3 agonists, such as mirabegron, are better tolerated by the older population, as well as in all age groups, and thus provides another option that is just as efficacious as antimuscarinic medications [24, 25], especially in the setting of combination therapy. Although hypertension and a history of palpitations are significant considerations when prescribing mirabegron, the significant potential for cognitive side effects of antimuscarinics do not apply to β-3 agonists and therefore support the use of mirabegron in the older population at this time. Therefore, although the current study validates and further amplifies the results of previous studies supporting combination therapy with medication plus peripheral neuromodulation, the contraindications and warnings regarding antimuscarinics further support the notion of starting with a β-3 agonist in combination with PTNS rather than combination therapy with antimuscarinic medications.

Strengths of the study include that this was a randomized controlled trial with the use of an oral placebo, and both participants and providers were blinded to the medication treatment allocation. It is important to note that the patients in this study had on average a very high mean number of UUI and SUI episodes per day, which is a population-specific characteristic for this study. Limitations include limited racial diversity and a single study site, which limits external generalizability. The attrition rate was noted to be slightly higher than estimated; however, it was less than an acceptable value of 20%, where bias related to attrition may occur [26]. Further, although inherent to the treatment, PTNS is provided over a 12-week in-person treatment regimen and can become burdensome to some patients. This accounted for the main reason for the 8 subjects not completing the 12-week PTNS treatment protocol. Other main reasons for not consenting, for withdrawing, or for dropping out included difficulty with obtaining transportation, parking difficulty, conflicting obligations, and financial constraints. With the current development of implantable PTNS options, this burden may be overcome in the near future [27, 28]. Finally, it is unclear whether combined therapy results in longer treatment effects.

After study completion, patients were offered continuation in their specified group of treatment. All patients were offered the opportunity to continue on PTNS alone, PTNS plus mirabegron, or mirabegron alone if they chose to no longer come for PTNS booster treatments. Last, although there is currently no follow-up study planned, follow-up studies looking at a combination of PTNS plus vibegron or implantable PTNS devices combined with either mirabegron or vibegron could be considered.

In conclusion, in subjects undergoing PTNS treatment for refractory UUI symptoms, the addition of the β-3 agonist, mirabegron, produced significant improvement in both objective and subjective overactive bladder symptom outcomes compared with PTNS plus placebo. These data should be considered and discussed with patients presenting to providers with refractory UUI symptoms considering PTNS therapy.
